# Epidemiological Analysis of the 2019 Dengue Epidemic in Bhutan

**DOI:** 10.3390/ijerph18010354

**Published:** 2021-01-05

**Authors:** Tsheten Tsheten, Angus Mclure, Archie C. A. Clements, Darren J. Gray, Tenzin Wangdi, Sonam Wangchuk, Kinley Wangdi

**Affiliations:** 1 Research School of Population, Australian National University, Acton, Canberra, ACT 2601, Australia; angus.mclure@anu.edu.au (A.M.); darren.gray@anu.edu.au (D.J.G.); kinley.wangdi@anu.edu.au (K.W.); 2 Royal Centre for Disease Control, Ministry of Health, Thimphu 11001, Bhutan; swangchuk@health.gov.bt; 3 Faculty of Health Sciences, Curtin University, Perth, WA 6102, Australia; archie.clements@curtin.edu.au; 4 Telethon Kids Institute, Nedlands, WA 6009, Australia; 5 Vector-Borne Disease Control Program, Ministry of Health, Gelephu 31102, Bhutan; karbay2014@gmail.com

**Keywords:** dengue, epidemic, Bhutan, dispersion, transmissibility

## Abstract

Bhutan experienced its largest and first nation-wide dengue epidemic in 2019. The cases in 2019 were greater than the total number of cases in all the previous years. This study aimed to characterize the spatiotemporal patterns and effective reproduction number of this explosive epidemic. Weekly notified dengue cases were extracted from the National Early Warning, Alert, Response and Surveillance (NEWARS) database to describe the spatial and temporal patterns of the epidemic. The time-varying, temperature-adjusted cohort effective reproduction number was estimated over the course of the epidemic. The dengue epidemic occurred between 29 April and 8 December 2019 over 32 weeks, and included 5935 cases. During the epidemic, dengue expanded from six to 44 subdistricts. The effective reproduction number was <3 for most of the epidemic period, except for a ≈1 month period of explosive growth, coinciding with the monsoon season and school vacations, when the effective reproduction number peaked >30 and after which the effective reproduction number declined steadily. Interventions were only initiated 6 weeks after the end of the period of explosive growth. This finding highlights the need to reinforce the national preparedness plan for outbreak response, and to enable the early detection of cases and timely response.

## 1. Introduction

Dengue is an emerging vector-borne disease with a rapidly expanding global distribution and an increasing intensity of transmission in affected regions [1]. Globally, an estimated 390 million dengue infections occur each year and nearly 4 billion people are at risk of the disease [2,3]. A global dengue pandemic began in South-East Asia and the Pacific region during and after World War II [4]. The movement of troops during World War II, along with the destruction of the environment and human settlement has substantially contributed to the spread of dengue viruses and their vectors in these regions [5]. Climate change, economic booms (with uncontrolled urbanization and development), and unprecedented human travel have contributed to further spread of the dengue epidemic, which continues to encroach on new geographical locations [5,6,7]. Before the 1970s, only nine countries experienced severe dengue epidemics [8]. However, dengue is now endemic in over 140 countries in Africa, the Americas, the Eastern Mediterranean, and Asia [9]. In the South-East Asia Region, severe dengue is the leading cause of hospitalization and death among children [10]. For these reasons, the World Health Organization (WHO) has included dengue in the potential public health emergency of international concern (PHEIC) list under the current International Health Regulations (IHR) [11].

Dengue is caused by arthropod-borne flaviviruses belonging to one or more of the four known dengue virus serotypyes (DENV1-4) [12]. The virus is transmitted through the bites of an infective female *Aedes* mosquitoes, mainly *Aedes aegypti*, and to a lesser extent, *Aedes albopictus*. Both species feed during the daytime from morning until dusk, although night-time biting is also reported in the case of *Ae. albopictus* [13]. *Ae. aegypti* breeds both indoors and outdoors, while *Ae. albopictus* is well-adapted to the peridomestic environment and typically breeds outdoors [14].

Bhutan is a Himalayan country, bordering China in the north and India in all other directions. The climate near the border with China does not support the survival of mosquitoes and hence, there is no transmission of dengue. Transmission of dengue is observed mainly along the border with India, particularly along the southern foothills, where the climate is subtropical. Bhutan reported its first dengue outbreak in Phuntsholing (a subdistrict of Chukha) in 2004 with more than 2500 cases. From 2013, dengue cases began to be reported in other districts along the border with India, where both vector species of *Aedes* are found [15]. Except for DENV-4, all other DENV serotypes have been reported, with frequent changes in the dominant serotype [16]. The DENV-3 epidemic of 2019 was Bhutan’s largest epidemic to date, with case numbers greater than the combined number reported in all previous years. At the peak of the epidemic, the government implemented a strategy of regular vector control, which included mass thermal fogging and public awareness messaging (on week 34), a mass cleaning campaign with a focus on larval source reduction (on weeks 34–37), travel advisory notifications to the general public (on week 35), and case-based indoor residual spraying and a postal health education campaign (on week 39).

Geographical information systems (GIS), combined with spatial analytical methods, have been increasingly utilized to collate, map, analyze, and interpret epidemiological information in relation to zoonotic and vector-borne diseases [17], and to facilitate better public health communication, collaboration, and decision making [18]. Spatiotemporal analysis utilizing GIS functionality can provide crucial information for control programs by allowing prompt assessment of patterns of the evolution of an outbreak. Additionally, the time-varying effective reproduction number (*Rt*) is used to quantify the transmissibility of infectious disease in a population over the course of an epidemic, and provides a measure of the strength of additional interventions required to halt the spread of a disease [19]. The transmission of vector borne diseases within a population depends on duration and degree of infectiousness, contact rate between vectors and hosts, and susceptibility of individuals. The combined effect of these variables are summarized by *Rt*, which is the average number of secondary cases produced by an index case over the course of its infectious period. In general, the number of cases will increase when *Rt* > 1 and decrease when *Rt* < 1 [20]. By maintaining *Rt* < 1, infectious diseases can be controlled and eradicated. Therefore, *Rt* can be used to estimate the intensity of control interventions necessary to contain an outbreak [21].

This epidemic provides an opportunity to analyze transmissibility and spatiotemporal patterns of emergence of dengue in real life situations where public health interventions are implemented simultaneously during outbreaks. The aim of this study was to quantify different spatial and temporal aspects of the dengue epidemic in Bhutan in 2019 to assist future epidemic management and planning. The objectives of the study were to explore the spatiotemporal characteristics of the epidemic and estimate the transmissibility of circulating dengue viruses.

## 2. Materials and Methods

### 2.1. Study Area

In Bhutan, elevation ranges from 160 m to more than 7000 m above sea level. The climate is humid and subtropical in the southern plains and foothills, temperate in the inner valleys of the central region, and cold in the northern Himalayas. There are four seasons in Bhutan. The dry spring season starts in early March and ends in late May. Summer weather commences in June, beginning with occasional showers, followed by heavy rain as it approaches the month of August. The rain continues in the Autumn season, which stretches from September to November. At higher elevations, this season is marked by bright sunny days and some early snowfalls. From December, winter sets in with frost covering much of the country, and elevations above 3000 m above sea level are covered with snow [22].

Bhutan is administratively divided into 20 districts or *dzongkhags*. The population density in these districts ranges from as low as 1 person/km^2^ in Gasa, to as high as 65 people/km^2^ in Thimphu. In 2017, the mean population per district was 37,084 and ranged from 4086 to 146,466 people [23]. These districts are further broken down to a total of 205 subdistricts or *gewogs*.

### 2.2. Data Sources

We used both districts and subdistricts as the spatial units, and weeks as the temporal unit of analysis. The temporal unit is consistent with the existing reporting framework of the National Early Warning, Alert, Response and Surveillance (NEWARS). NEWARS is an integrated online disease surveillance system that collates data on all national notifiable diseases across Bhutan. All dengue cases are required to be reported into NEWARS from all health centers every week, while severe dengue or dengue hemorrhagic fever (DHF) has to be reported on the day of diagnosis. Dengue fever cases are defined as any patients with a history of travel or living in dengue-endemic areas presenting with fever and any two of the following symptoms: headache, retro-orbital pain, myalgia, arthralgia, rash, positive tourniquet test, or any warning signs of severe dengue (abdominal pain, persistent vomiting, mucosal bleeding, fluid accumulation, liver enlargement, and increasing hematocrit). Severe dengue is characterized by one or more of the following: positive tourniquet test, petechiae/ecchymoses/purpura, bleeding (mucous, GI, or other), thrombocytopenia, or evidence of plasma leakage [11]. Serological confirmation of the cases was conducted using NS1 antigens and/or IgM/IgG antibodies in the respective health center using rapid test kits provided by the Ministry of Health. Both dengue fever and severe dengue cases were combined for this analysis as the numbers of severe dengue cases were small (Supplementary Table S1).

Population estimates used in the study were obtained from the recent 2017 Population and Housing Census of Bhutan [23]. The full epidemic was considered for the study in which the number of cases started to rise, peaked, and decreased to nonepidemic levels. This period ranged from week 18 (29 April) to week 49 (8 December).

### 2.3. Descriptive Analysis

The overall cumulative incidence was calculated for each district and subdistrict by dividing the total number of dengue cases reported by health centers located in that district and subdistrict by their respective population, multiplied by 10,000. The ages of the cases were categorized into four groups as follows: <9 years, 10–19 years, 20–49 years, and ≥50 years. These age categories were presented as a proportion of total cases in each district.

To visualize the changing spatial and temporal patterns of the dengue epidemic, separate maps were created at the subdistrict for four different time periods: weeks 18–25, 26–28, 29–34, and 35–49. The intervals between the time periods were created on the basis of the distribution of cases as per the phases of the epidemic curve explained in a supplementary analysis (Supplementary Material S1). The areas were classified as “newly affected”, “ever affected”, and “nonaffected” areas. “Newly affected” areas were defined as those subdistricts that have reported dengue for the first time during the time period, and these subdistricts were designated as “ever affected” in the subsequent time periods.

### 2.4. Growth Rate

The number of new cases of a disease often grows exponentially during the early stages of an epidemic and declines exponentially at the end of an epidemic. The exponential growth rate *r*—i.e., the per capita increase or decrease in the number of new cases per unit time—is a useful measure of the growth trajectory of an epidemic. Inspection of the number of new cases reported each week over the course of the epidemic revealed four distinct phases in the epidemic. We estimated the value of the growth rate, *r*, for different stages of the epidemic by fitting a linear model to the log of the number of new cases reported each week during that phase, using the function *glm* from the *stats* R package [24].

### 2.5. Effective Reproduction Number

The effective reproduction number, *Rt*, for a vector borne disease is defined as the mean number of secondary cases arising from a typical primary case. Like the growth rate, the value of *Rt* can change over the course of an epidemic due to changes in environmental factors, human behaviors, healthcare responses, and the proportion of people with immunity to the disease.

When it is reasonable to assume that the serial interval, defined as the time between symptom onset in a primary case and a secondary case, does not change over the course of an epidemic, one can estimate the effective reproduction number as a simple function of the growth rate and the serial interval. We use such a method in a supplementary analysis (see Supplementary Material S1). However, as the length of the incubation period in mosquitoes is temperature-sensitive and therefore seasonally variable, for our primary analysis we estimated the time-varying, temperature-dependent cohort reproduction number using the method developed by Codeco et al. [25], which is a temperature-dependent extension of the method proposed by Wallinga and Teunis [26,27]. In brief, the method proposed by Wallinga and Teunis, which was first applied to SARS, used the distribution of the serial interval and the number of cases reported in each reporting period (e.g., each day or each week) to estimate for each case, the relative likelihood that they were infected by each case in the preceding reporting periods. The effective reproduction number for each reporting period was then estimated as the average number of cases thus attributed to each case in that reporting period. Codeco et al. extended this method to temperature-dependent vector-borne diseases by using observations of average daily temperature and the relationship between temperature and the extrinsic incubation period, to account for changes to the serial interval over time. They modeled the serial interval (measured in days) as the (truncated) sum of four independent periods: the intrinsic incubation period (gamma distributed with shape 16 and rate 2.7), the extrinsic incubation period (gamma distributed with shape 4.3 and rate 7.9 + 0.21 T, where T is the average temperature in degrees Celsius), infectious period in humans and infection period in mosquitoes (exponentially distributed with mean 1). The serial interval distribution is truncated at 5 weeks, to account for the short lifespan of vectors.

We adapted this method and the published R code by Codeco et al. for our analysis. We calculated the time-dependent effective reproduction number for the whole country (i.e., using cases reported in all districts), and for Chukha only. Since cases were concentrated in Chukha district and the climatically similar southern districts, we used the (loess smoothed) weekly average daily temperature in Chukha to inform the temperature-dependent model of the extrinsic incubation period. To assess the effect of temperature-dependence on our estimates of the reproduction number, we also made temperature independent estimates of the effective reproduction number by adopting the counterfactual assumption that temperature was fixed at the average value across the epidemic period. As a sensitivity analysis exploring the effect of under-reporting in the early weeks of the epidemic, we calculated the temperature dependent time-varying reproduction number for Bhutan under the assumption that the proportion of dengue infections reported to NEWARS was up to 80% lower in the early weeks of the epidemic than the remainder of the epidemic (Supplementary Material S2).

## 3. Results

### 3.1. Descriptive Analysis

**Figure 1:**
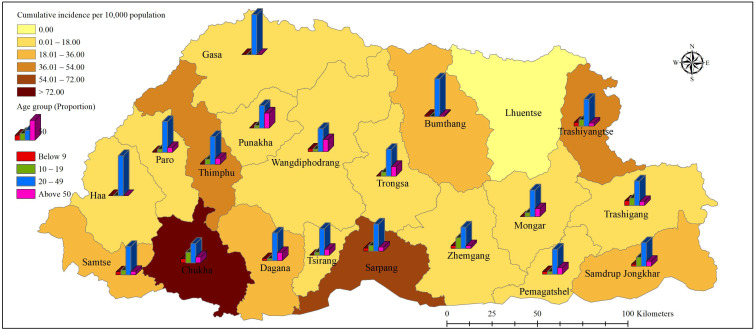
Distribution of cumulative incidence and proportion of cases by age groups as reported by health centers in each district during the dengue epidemic period in Bhutan, from weeks 18–49. The height of the bar corresponds to the proportionality of four age groups among dengue cases in each district.

**Figure 2:**
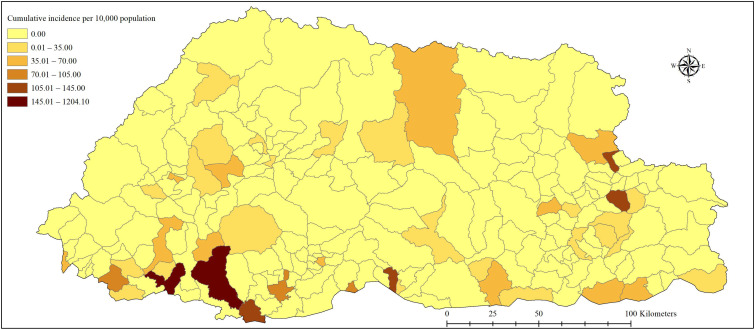
Distribution of cases by cumulative incidence by subdistrict during dengue epidemic in Bhutan, from weeks 18–49. Subdistricts with the darker color include Phuntsholing, Bongo, Gelephu, Lhamozingkha, and Khamdang subdistricts. Khamdang subdistrict in the top central-eastern region saw a cluster of cases (≈70 cases) for the first time in this epidemic.

During the study period, 5935 cases of dengue were reported across the country. Of this, 26 (0.49%) cases were related to severe dengue. The epidemic curve showed a steep rise in cases from weeks 26 to 28 (Supplementary Figure S1). The overall cumulative incidence for the entire country was 80.02 cases per 10,000 population. Chukha was the worst-affected district with a cumulative incidence of 607.55 cases per 10,000 population. Sarpang, Thimphu and Trashiyangtse followed next with ≈40–60 per 10,000 population (Figure 1). At the subdistrict level, Phuntsholing, Bongo (in Chukha), Gelephu (in Sarpang), Lhamozingkha (in Dagana), and Khamdang (in Trashiyangtse) reported the highest incidences with 1204.1, 211.9, 137.3, 127.0, and 114.4 per 10,000 population, respectively (Figure 2). The maximum proportion was reported in the age group of 20–49 years in all districts followed by the 10–19 years age group (Figure 1). The overall proportion of age groups <9, 10–19, 20–49, and ≥50 years were 8.27%, 22.30%, 55.00%, and 14.43%, respectively (Table 1).

**Figure 3:**
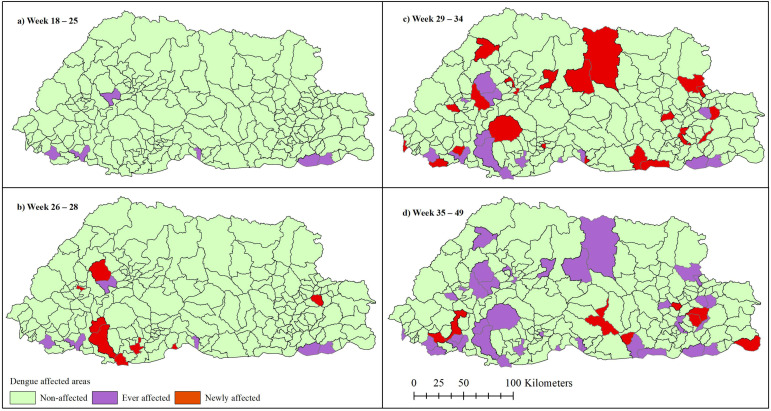
The changing spatiotemporal pattern of 2019 dengue epidemic in Bhutan at the subdistrict level. The epidemic initially started with six subdistricts as shown in the first panel in the top-left map (**a**). These subdistricts have also reported dengue in the past. The epidemic spread to 8, 22, and 8 new subdistricts in weeks 26–28 (**b**), 29–34 (**c**), and 35–49 (**d**), respectively, that summed to 44 subdistricts at the end.

Only six subdistricts had ever reported cases before the 2019 epidemic. The cumulative number of newly affected subdistricts increased rapidly from zero in weeks 18–25 to eight in weeks 26–28, 22 in weeks 29–34 and eight in weeks 35–49. In total, 44 subdistricts were affected resulting in 5935 cases across a span of 32 weeks (Figure 3).

### 3.2. Growth Rate

There were four apparent phases to the epidemic (Supplementary Figure S2, Supplementary Table S2). In the initial phase (weeks 18–25) the number of cases reported was <10 per week and the growth rate was slow (*r* = 0.16; 95%CI: −0.01–0.33). The second phase (weeks 25–28) was a short period of explosive growth (*r* = 1.35; 95%CI: 0.99–1.71), by the end of which there were >300 new cases reported each week. In the third phase (weeks 28–34) case numbers remained high, but growth slowed (*r* = 0.12; 95%CI: 0.07–0.17) to levels similar to phase 1. In the final phase (weeks 34–49), case numbers declined (*r* = –0.29; 95%CI: –0.32––0.27).

### 3.3. Effective Reproduction Number

**Figure 4:**
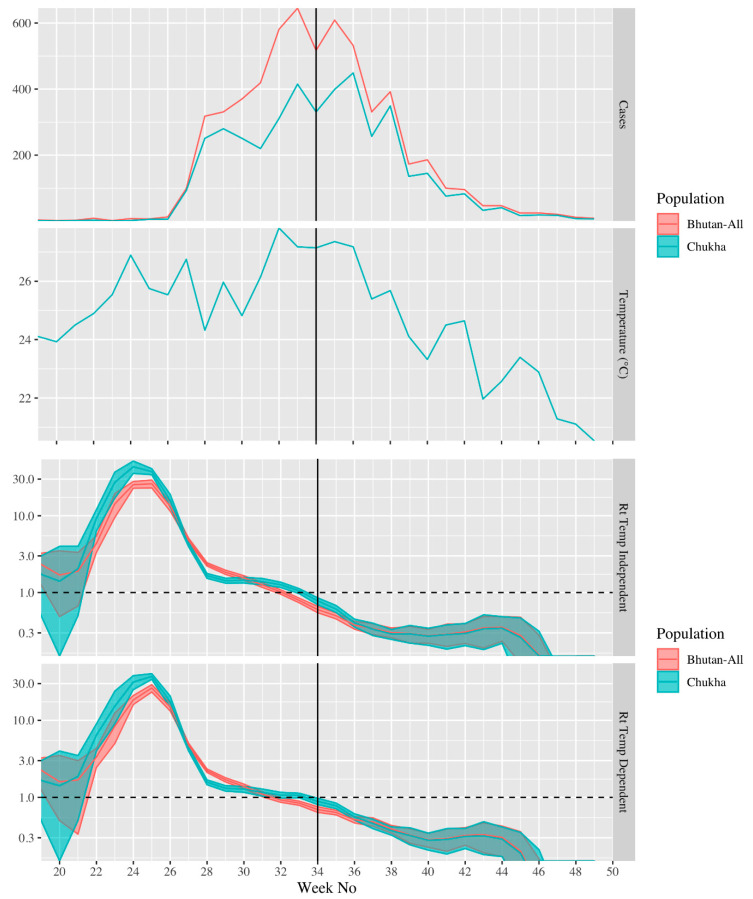
From top to bottom: the number of dengue cases reported in National Early Warning, Alert, Response and Surveillance (NEWARS) each week in all of Bhutan and Chukha only; the weekly average temperature (°C) in Chukha; the effective reproduction number for cases developing symptoms in each week, assuming constant temperature over the period; the effective reproduction number for cases developing symptoms in each week, accounting for changes in temperature over the epidemic. The solid vertical line indicates the start of interventions in week 34. The dashed horizontal line in the bottom two panels indicates the critical value of the effective reproduction number (1.0). In the bottom two panels, the shaded regions indicate 95% confidence intervals and the *y*-axis is on a log-scale for clarity.

The estimate of the effective reproduction number varied dramatically over the period (Figure 4). It was highest for cases that developed symptoms around epi-week 25, peaking at ≈30; however, the effective reproduction number declined sharply, reaching ≈1 in cases that developed symptoms around week 33 and continued to decline after this point (Figure 4). The estimates of the effective reproduction number for Chukha-only and all of Bhutan exhibited the same temporal pattern; however, the reproduction was generally higher in the Chukha-only model and became <1 later in the Chukha-only model (week 34) than in the Bhutan model (week 32) (Figure 4). The weekly average temperature in Chukha ranged from 24 to 27 °C between weeks 18 and 39. The temperature-dependent and temperature-independent estimates of the effective reproduction number were largely the same; though in the Chukha-only models the peak effective reproduction number was somewhat higher (43 vs. 37) and earlier (week 24 vs. week 25) than in the temperature independent model (Figure 4). The alternate visualization of effective reproduction over the course of the epidemic is presented in Supplementary Figure S3. The peak value of *Rt* was sensitive to potential under-reporting. For instance, if we assumed that the reporting proportion (i.e., proportion of dengue infections reported to NEWARS) before week 27 was only 80%, 40%, or 20% of the proportion in week 27 and onward, the peak estimate of temperature-dependent *Rt* was reduced to 21 (95% CI: 19–24), 12 (95% CI: 10–13), or 6.2 (95% CI: 5.5–6.8), respectively, compared to 26 (95% CI: 23–29) when we assumed the reporting proportion was constant over the whole period (Supplementary Figures S4 and S5).

We were unable to use our methodology to measure the effectiveness of the wide-scale interventions initiated in week 34. However, we note that the wide-scale interventions began ≈6 weeks after the end of the period of rapid growth in case numbers and ≈10 weeks after the peak in the effective reproduction number (Figure 4). Similarly, we note that our estimates of the time-varying reproduction number are primarily descriptive in nature; we did not use our method to infer the underlying causes of variability. The relatively minor differences between temperature-dependent and temperature independent estimates do not imply that temperature had a minor or major role in dengue transmissibility during the epidemic.

## 4. Discussion

The 2019 epidemic was the first nation-wide dengue epidemic in Bhutan. The epidemic initially spread from low-lying areas in the southern foothills and diffused to other parts of the country, reaching 19 of the 20 districts at the end of the epidemic. There were 38 newly affected subdistricts during this epidemic in addition to the six previously-affected subdistricts. The estimate for the reproduction number was <3 for most of the epidemic period, except for a 1-month period of explosive growth at the start of the epidemic during which the reproduction number peaked >30.

An explosive increase in cases, and very high reproduction number was observed between weeks 25 and 28, coinciding with the monsoon season which is characterized by increased rainfall and temperatures across the country (Supplementary Figure S6). Other countries have also observed increasing numbers of dengue cases during monsoon and warm seasons [28,29,30]. Rainfall increases the availability and productivity of breeding sites for female mosquitoes to lay eggs, and development of immature mosquitoes. Humidity maintains a conducive environment for the survival of adult mosquitoes. Humidity affects flight and host-seeking behavior, and lifespan of vectors [30]. The hot climate promotes dengue virus transmission by accelerating the biting rate, shortening the gonotropic cycle and reducing the extrinsic incubation period [31].

An unprecedented geographical expansion of dengue was observed in the 2019 epidemic, expanding from six subdistricts with a history of sporadic cases of dengue outbreaks to a total of 44 (total of 205) subdistricts during this outbreak. The incidence among the affected subdistricts ranged from 1.8 to 1204.1 per 10,000 population. Human movement may have played an important role in the spread of dengue infection. Firstly, the outbreak season coincided with the school holidays which is generally associated with increased travel of students and parents from schools to their residential subdistricts and vice versa after the school holidays. Secondly, a majority of the cases were people aged 20–49 years. People in this age range are involved in economic activities and often travel from their subdistrict of residence to other subdistricts. For instance, people from other parts of the country travel to Phuentsholing Town located in Phuentsholing subdistrict to sell their farm produce and for other business purposes. Similarly, most of the goods and commodities entering Bhutan are ferried through this town. Historically and during this outbreak, Phuentsholing remained the epicenter of dengue epidemic. It is plausible for the people from other subdistricts to have got the infection from Phuentsholing during one of such visits and imported it to their subdistricts.

Local transmission in subdistricts is possible because *Ae. aegypti* had been reported in Chukha, Samtse, Samdrup Jongkhar, and Wangdiphodrang, while *Ae. albopictus* is present in 16 districts excepting Thimphu, Gasa, Bumthang, and Paro [15]. In addition, *Ae aegypti* was detected in Khamdang subdistrict in Trashiyangtse district during this epidemic for the first time. Kraemer and colleagues have shown that even under current climatic conditions, continuous human movement will enable dengue vectors to spread and occupy suitable habitats, posing a risk to human health [32]. *Ae. aegypti* tend not to disperse far from households because human blood sources, mates, and places to lay their eggs are abundant around homes where they reside [33]. The limited flight range of mosquitoes means that rapid geographic spread of dengue during epidemics is more likely driven by the movement of viremic humans rather than the flight of infected mosquitoes [33].

Our results suggest that the dengue epidemic in 2019 consisted of four phases. After 3 weeks of explosive growth and 6 weeks of moderate transmission and consistently high case numbers, incidence peaked around week 34, coinciding with the beginning of interventions. However, decreasing rainfall and temperatures (Supplementary Figure S6), and naturally acquired immunity in the human population may have accounted for the reduction in transmission. Nevertheless, the size of the epidemic may have been smaller if these interventions were applied earlier, for instance around weeks 25–28 after the rapid growth of cases. However, late initiation of public health responses when the transmission is near its peak, or has started to decline, is a common issue in many countries [34].

We estimated that the effective reproduction number was approximately <3 for most of the epidemic prior to the onset of interventions, except for a 1-month period of explosive growth approximately one serial interval before the rapid rise in cases numbers, where the effective reproduction number was >10. Our estimate of the effective reproduction number in the periods of slower growth is similar to estimates of the basic reproduction number in some countries, such as Singapore (*R_0_*≈1.89 to 2.23, 2005) [34], Taiwan (*R_0_*≈3.90 to 4.60, 2007) [35], and Mexico (*R_0_*≈2.00–3.09, 2002) [21]. The effective reproduction number from the period of explosive growth in our study falls within the range basic reproduction number estimates of studies conducted in other countries such as Chile (*R_0_*≈27.20, 2002) [36], Brazil (*R_0_*≈2.0–103, 1996–2003) [37], and Indonesia (*R_0_*≈8.00–39.45, 2002–2007) [38].

Except DENV-4, the circulation of other three DENV serotypes varied over time in Bhutan. DENV-2 was the predominant serotype in 2004, while DENV-2 and 3 predominated in 2005 and 2006 [16]. A serotype shift with predominant DENV-1 was reported in 2013–2014, and DENV-1 and 2 in 2016–2017 [15,39]. After an absence of more than a decade, DENV-3 re-emerged and predominated in the current outbreak. As reported in other countries [40,41,42], the intense transmission and higher incidence in this outbreak might be related to a shift in the predominant serotype. Further, DENV-3 is often associated with intense force of transmission [43]. Previous outbreaks in Bhutan have been linked to the importation of dengue virus from neighboring countries in the South-East Asia region [16,39]. As the surveillance system did not capture information on the travel history of the dengue patients, the source that triggered this large epidemic could not be established. Phylogenetic analysis of the viral isolates might add this information by indicating the similarity of strains with other neighboring countries.

Estimates of basic and effective reproduction numbers can be sensitive to the type of methods used [21], complicating the comparison across studies that use different methods. Nevertheless, estimates of the effective reproduction number around 30 are remarkably high. Codeco et al. have shown that failing to account for changes in temperature can lead to the overestimation of effective reproduction numbers in the early stages of seasonally driven epidemics [25]. However, using their method, we found that the peak effective reproduction number remained high even after accounting for temperature changes. Moreover, in an alternative analysis in which we calculated the effective reproduction number from the growth rate in the four phases of the epidemic (see Supplementary Material S1), the peak estimated value of the reproduction number was also high (31; 95% CI 15–61).

The unprecedented size and geographic extent of the epidemic and the delay between the rapid rise in cases and implementation of interventions highlight the need to strengthen the existing surveillance and response mechanisms in Bhutan. Given the late initiation of dengue interventions in the 2019 outbreak, effort should be undertaken to develop a more rapid response, including the possible development and use of early warning systems, for future years.

Our study is subjected to several limitations. The data were obtained from the national surveillance database and it is likely that people with mild or asymptomatic illness did not seek care in public health facilities and were not captured by the surveillance system. Moreover, as dengue is a nonspecific febrile illness, diagnosis may be less likely when the clinical suspicion is low, such as in the early stages of epidemics and in regions with no history of dengue infection. Consequently, the completeness of cases in the database may have varied across the epidemic. In particular, if the rapid rise in reported case numbers observed in weeks 25–27 were in part due to increasing public awareness of healthcare-seeking behavior, then we will have overestimated the true reproduction number and growth rate in the early stages of the epidemic. Travel history of the cases was not captured by the surveillance system. As dengue has an intrinsic incubation period of 3–14 days [1], without this travel information, we were not able to rule out the acquisition of cases outside Bhutan or determine with certainty the region within Bhutan in which the infection was acquired. Moreover, the total number of cases in many districts was low and the center of the epidemic (Chukha) was a national travel and commerce hub. We were therefore unable to estimate growth rates and reproduction numbers for every district. The reproduction number may have been much lower in districts with lower densities of dengue vectors and humans.

## 5. Conclusions

This study reports the spatiotemporal patterns and effective reproduction number of the first national dengue epidemic in Bhutan. Dengue was reported by 19 of the 20 districts in the country. A very high transmissibility of dengue virus was found, with a high reproduction number during a period of explosive growth. This finding highlights the need to reinforce the national preparedness plan for outbreak response, and to enable the early detection of cases and timely response.

## Figures and Tables

**Table 1:** Proportion of affected dengue cases by age categories during the dengue epidemic in 2019, Bhutan (note: 688 records do not have information on age).

Age Categories (Years)	Number of Cases (*n*)	Proportion (%)
0–9	434	8.27
10–19	1170	22.30
20–49	2886	55.00
≥50	757	14.43

## Data Availability

Data supporting the findings of the study is available in the supplementary material (Table S1).

## Supplementary Materials

The following are available online at https://www.mdpi.com/1660-4601/18/1/354/s1. Supplementary Table S1: Dataset showing weekly and cumulative dengue cases of the 2019 dengue epidemic in Bhutan, Supplementary Table S2: Growth rate and reproduction number during phases of the dengue epidemic in Bhutan, 2019, Supplementary Material S1: Supplementary analysis, Supplementary Material S2: Sensitivity analysis, Supplementary Figure S1: Epidemic curve showing weekly number of new cases (left vertical axis) along with the weekly cumulative cases of dengue epidemic in Bhutan in 2019, Supplementary Figure S2: Epidemic curve showing the different growth rates across the four phases of the epidemic. The weekly reported cases numbers collected by NEWARS (National Early Warning, Alert, Response and Surveillance) are represented by dots, the fitted exponential growth curves and 95% confidence intervals are indicated by lines and shaded regions. The transition between phases one and two (week 25) coincided with the beginning of high rainfall and the start of school vacation. The transition between phases three and four (week 34) coincides with the beginning of national interventions, Supplementary Figure S3: The effective reproduction number over the course of the epidemic, assuming constant temperature and accounting the changes in temperature in all of Bhutan and Chukha only. Supplementary Figure S4: Sensitivity analysis to account the influence of under-reporting on the reproduction number by considering cut-offs in weeks 25, 26, 27, and 28, and reporting ratios of 0.2, 0.4, 0.6, 0.8, and 1.0. Supplementary Figure S5: The influence of under-reporting at the beginning phase of the epidemic on the estimated temperature dependent, time varying reproduction number for the whole country. Supplementary Figure S6: Total weekly rainfall and average temperature in Bhutan, 2019. Multiyear averages for these variables were calculated using data from 2008 to 2018. (Source: National Centre for Hydrology and Meteorology of the Royal Government of Bhutan).
