# Supplementary material for: Epidemiological Analysis of the 2019 Dengue Epidemic in Bhutan
Source: Int J Environ Res Public Health. 2021 Jan 5;18(1):354. doi: 10.3390/ijerph18010354 (PMC7796457; doi:10.3390/ijerph18010354)
Supplement: Supplementary file 1 [file ijerph-18-00354-s001.zip › ijerph-1026975-Table S6.docx]

**Table S6.** Growth rate and Reproduction number during phases of the dengue epidemic in Bhutan, 2019.

| **Week number** | **Growth rate, *r* (95% CI)** | **Reproduction number, *R* (95% CI)** |
| --- | --- | --- |
| 18 – 25 | 0.16 (-0.01, 0.33) | 1.8 (0.95, 3.26) |
| 25 – 28 | 1.35 (0.99, 1.71) | 31 (15.20, 60.70) |
| 28 – 34 | 0.12 (0.07, 0.17) | 1.5 (1.30, 1.92) |
| 34 – 49 | -0.29 (-0.32, -0.27) | 0.22 (0.16, 0.29) |
