# Supplementary material for: Epidemiological Analysis of the 2019 Dengue Epidemic in Bhutan
Source: Int J Environ Res Public Health. 2021 Jan 5;18(1):354. doi: 10.3390/ijerph18010354 (PMC7796457; doi:10.3390/ijerph18010354)
Supplement: Supplementary file 1 [file ijerph-18-00354-s001.zip › Supplementary material S2.docx]

**Supplementary analysis S2**

As a supplementary analysis we estimate the reproduction number from the growth using the formula:

$$Rt=\left( 1+r \tau_{H} \right)\left( 1+r \tau_{M} \right)\left( 1+r \tau_{e} \right)\left( 1+r\tau_{i} \right).$$

where $\tau_{e}$is the mean extrinsic incubation period, $\tau_{i}$ is the mean intrinsic incubation period, $\tau_{H}$ is the mean duration of viremia in humans, and $\tau_{M}$is the mean duration of viremia in mosquitoes, which is assumed to be the expected additional life of the vector. Other authors have used the formulae $Rt=e^{r(\tau_{e}+\tau_{i})}$ or $Rt={\left( 1+r \tau_{H} \right)\left( 1+r \tau_{M} \right)e}^{r(\tau_{e}+\tau_{i})}$[1]. However, the first formula fails to account for the duration of viremia, while both formulae implicitly assume that the incubation period is exactly$\tau_{i}$ for all human cases and that the incubation period is exactly$\tau_{e}$ for all mosquitos; an assumption that tends to over-estimate the reproduction number [2].

Inspection of the number of new cases reported each week over the course of the epidemic revealed four distinct phases in the epidemic. We estimated the value of the growth rate, $r$, for different stages of the epidemic by fitting a linear model to the log of the number of new cases reported each week during that phase, using the function *glm* from the *stats* R package [3]. For the purposes of these estimates, the four phases were defined with three breakpoint weeks which were considered the last week of the preceding phase and the first week of the following phase. The breakpoint between phases one and two (week 25) was chosen to correspond to the start of summer school vacation and a period of high rainfall (i.e. the monsoon season). The breakpoint between phases three and four (week 34) was chosen to correspond to the beginning of large-scale interventions. The breakpoint between stages two and three (week 28) was chosen by inspection. We confirmed our choice of breakpoints by using the function *segmented*, from the R package, segmented [4], to fit a piecewise log-linear model and estimate the choice of the breakpoints. The breakpoint estimates using this procedure were week 25.7 (95% CI 25.2–26.3), week 27.6 (95% CI 26.9–28.2) and week 35.2 (95% CI 33.9–36.6). Moreover, the best fit model with four phases had a lower Akaike information criterion (26.2) than the best fit with three phases (53.4), while models with five or more phases failed to converge, confirming the suitability of a four-phase model. Breakpoints at 25, 28, and 34 were preferred as these aligned with the weekly reporting structure of the NEWARS data, provided a more robust estimate of the growth rate in phase two by including two additional data points, and allowed for the comparison of growth rates before and after interventions.

The estimates for the growth rate ($r$) in each phase was combined with estimates of $\tau_{H}, \tau_{M,} \tau_{e,}{and \tau}_{i}$ drawn from the literature [Table 1 in [5]], to estimate the reproduction number for each period. We used Monte Carlo sampling from each parameter’s uncertainty distribution to calculate confidence intervals for the reproduction number. We modelled the uncertainty in the growth rate, $r$, using a normal distribution with mean and standard deviation determined by the maximum likelihood estimate and standard error. Following Enduri and Jolad [5] we used gamma distributions to model the uncertainty in the remaining parameters: lifespan of vector (mean 8.88 days; s.d. 1.33 days) [6], duration of viremia (mean 5 days; s.d. 1 day) [6], extrinsic incubation period (mean 10 days; s.d. 1 day) [7], and intrinsic incubation period (mean 5.38 days, s.d. 0.73 days) [7]. By drawing 10^7^ sets of parameters from these distributions and calculating $Rt$ for each set, we calculated the median and 95% highest density intervals using the package *coda* in R [8].

Using this method, the estimated reproduction number was approximately the same in phases one and three with $Rt=1.8$ (95% CI 0.95–3.26) and $Rt=1.5$ (95% CI 1.30–1.92) respectively. However, the reproduction number was very high in phase two ($Rt=31.0$, 95% CI 15.2–60.7) and less than one in phase four ($Rt=0.22$, 95% CI 0.16–0.29) (Supplementary Table S6).

**References**

1. Favier C, Degallier N, Rosa-Freitas MG, Boulanger JP, Costa Lima JR, Luitgards-Moura JF, et al. Early determination of the reproductive number for vector-borne diseases: the case of dengue in Brazil. Trop Med Int Health. 2006;11(3):332-40. doi: 10.1111/j.1365-3156.2006.01560.x.

2. Wallinga J, Lipsitch M. How generation intervals shape the relationship between growth rates and reproductive numbers. Proc Biol Sci. 2007;274(1609):599-604. doi: 10.1098/rspb.2006.3754.

3. R Core Team. R: A Language and Environment for Statistical Computing: R Foundation for Statistical Computing, Vienna, Austria; 2019.

4. Muggeo VMR. Interval estimation for the breakpoint in segmented regression: a smoothed score‐based approach. Aust N Z J Stat. 2017;59(3):311-22.

5. Enduri MK, Jolad S. Estimation of reproduction number and non stationary spectral analysis of dengue epidemic. Math Biosci. 2017;288:140-8. doi: 10.1016/j.mbs.2017.03.007.

6. Chowell G, Fuentes R, Olea A, Aguilera X, Nesse H, Hyman JM. The basic reproduction number R0 and effectiveness of reactive interventions during dengue epidemics: the 2002 dengue outbreak in Easter Island, Chile. Math Biosci Eng. 2013;10(5-6):1455-74. doi: 10.3934/mbe.2013.10.1455.

7. Gubler DJ. Dengue and dengue hemorrhagic fever. Clin Microbiol Rev. 1998;11(3):480-96.

8. Plummer M, Best N, Cowles K, Vines K. CODA: convergence diagnosis and output analysis for MCMC. R news. 2006;6(1):7-11.
