# Supplementary material for: Epidemiological Analysis of the 2019 Dengue Epidemic in Bhutan
Source: Int J Environ Res Public Health. 2021 Jan 5;18(1):354. doi: 10.3390/ijerph18010354 (PMC7796457; doi:10.3390/ijerph18010354)
Supplement: Supplementary file 1 [file ijerph-18-00354-s001.zip › Supplementary material S3.docx]

**Supplementary analysis S3**

Public awareness, treatment seeking behaviour and clinical suspicion of dengue may have varied over the period. This may have led to different reporting rates over the study period. We considered a sensitivity analysis in which the proportion of cases reported in the early part of the epidemic was lower than for the remainder of the period. We considered cut-offs in weeks 25, 26, 27 and 28, and reporting ratios of 0.2, 0.4, 0.6, 0.8 and 1.0. For each combination of cut-off week and reporting ratio, we multiplied the number of cases reported in all weeks prior to the cut-off by the inverse of the reporting ratio (Supplementary Figure S8) and estimated the temperature dependant, time varying reproduction number for the whole country (Supplementary Figure S9) using these inflated case numbers. Assuming lower reporting ratios in the early weeks of the epidemic reduced the estimates of the reproduction number five weeks either side of the cut-off, and reduced the estimated peak value of the reproduction number. For a given reporting ratio, a cut-off at week 27 led to the lowest estimates of time-varying reproduction numbers.
