# Supplementary figures and images for: Epidemiological Analysis of the 2019 Dengue Epidemic in Bhutan
Source: Int J Environ Res Public Health. 2021 Jan 5;18(1):354. doi: 10.3390/ijerph18010354 (PMC7796457; doi:10.3390/ijerph18010354)

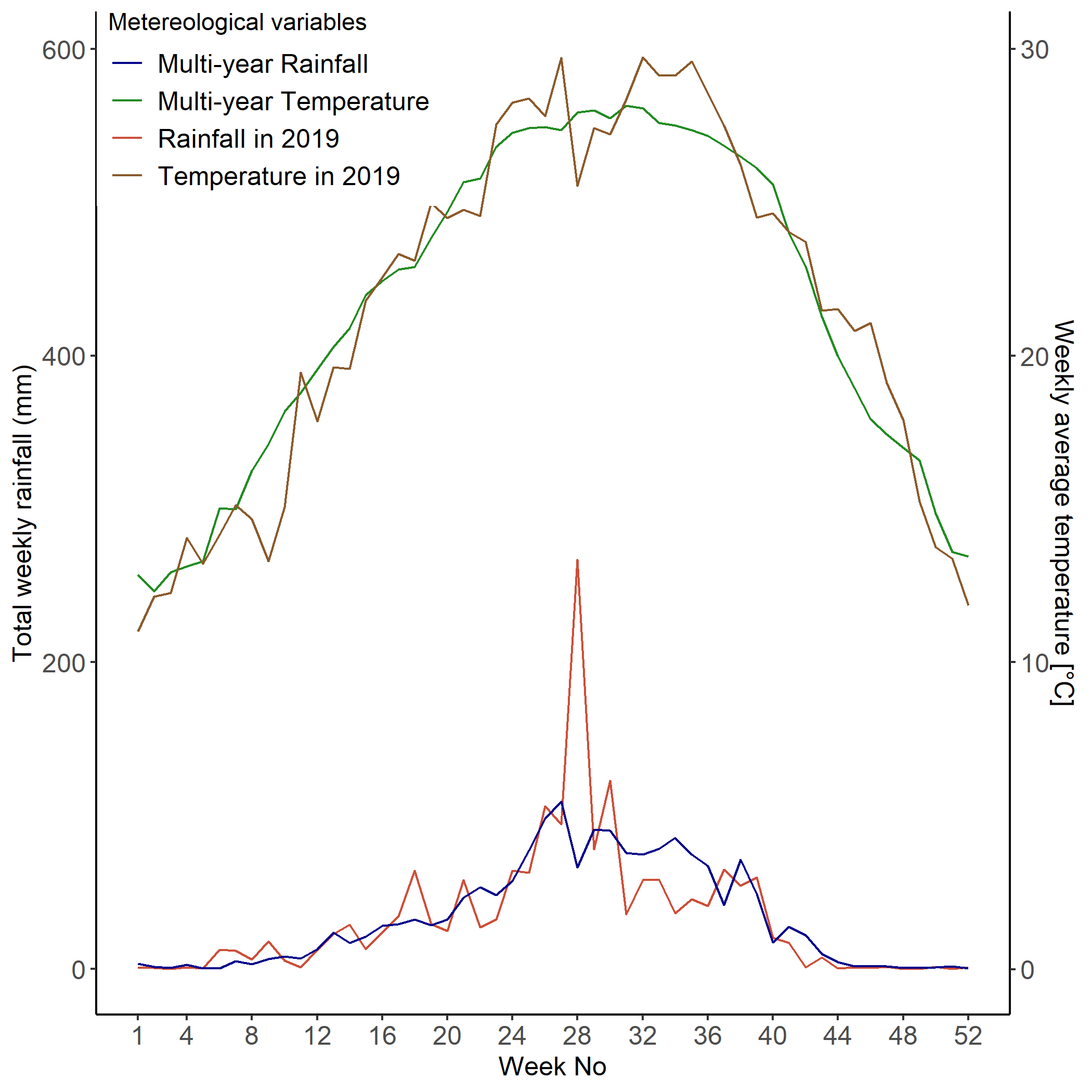

Supplement: Supplementary file 1 [file ijerph-18-00354-s001.zip › Supplementary Figure S10.tiff]

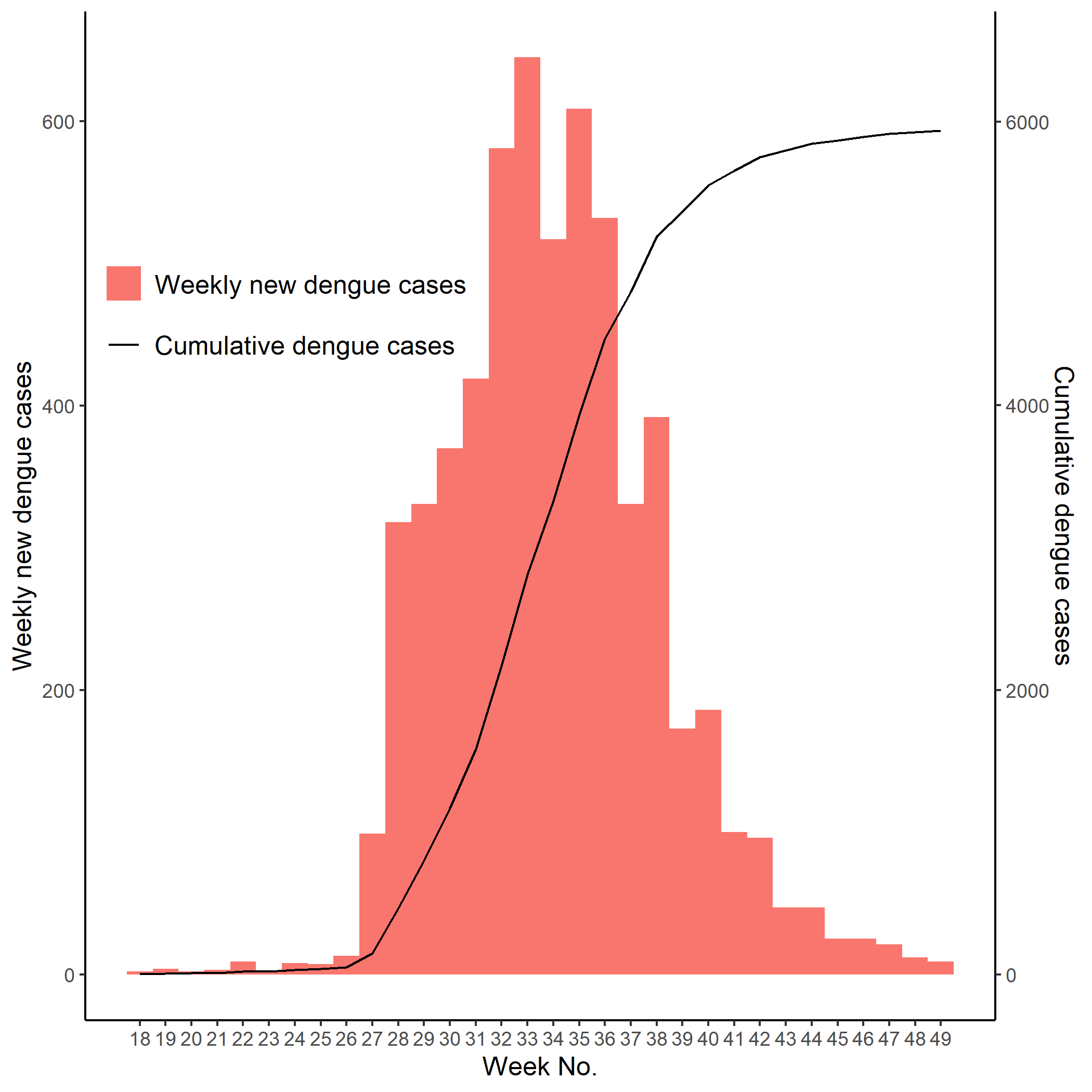

Supplement: Supplementary file 1 [file ijerph-18-00354-s001.zip › Supplementary figure S4.tif]

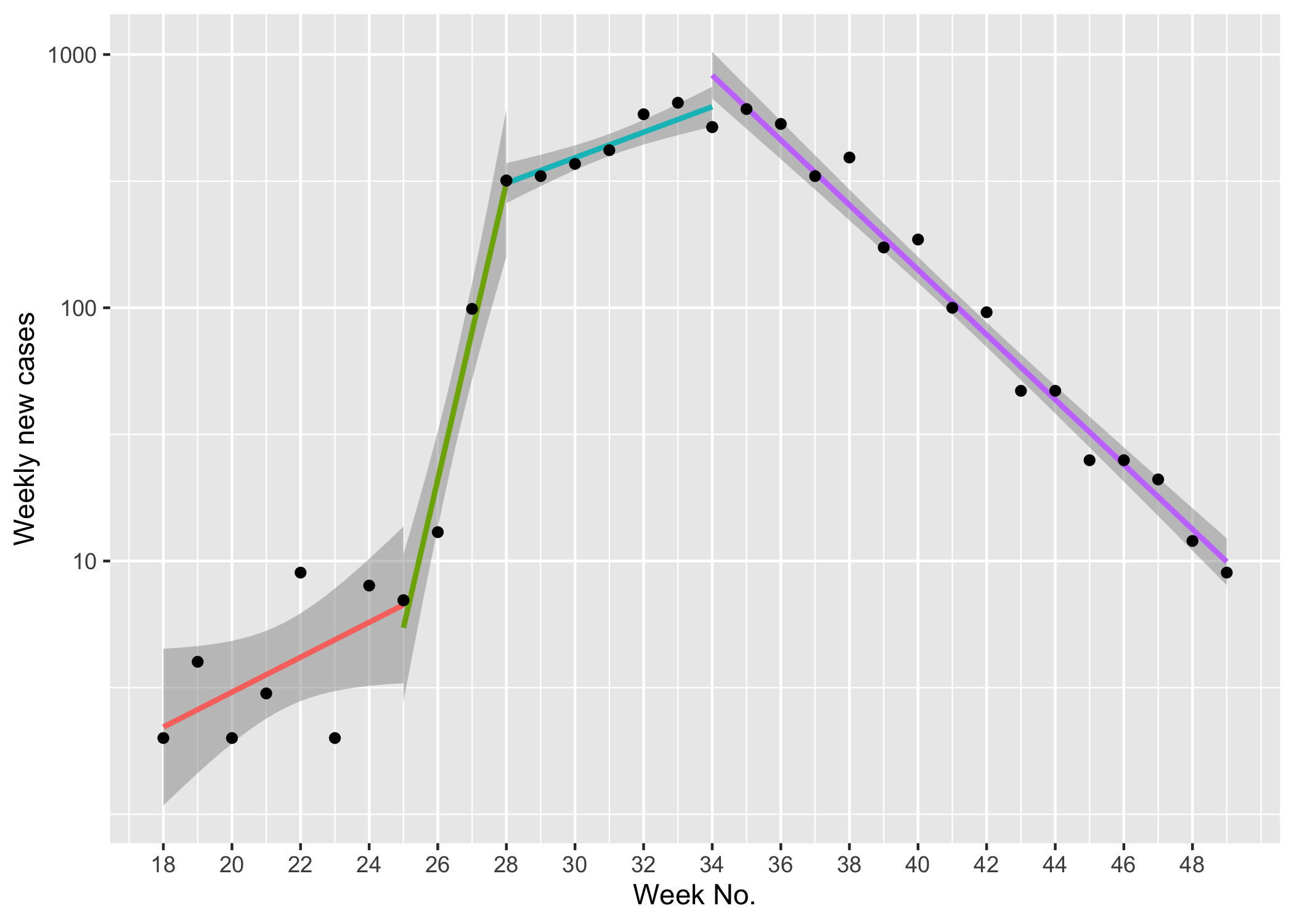

Supplement: Supplementary file 1 [file ijerph-18-00354-s001.zip › Supplementary figure S5.tif]

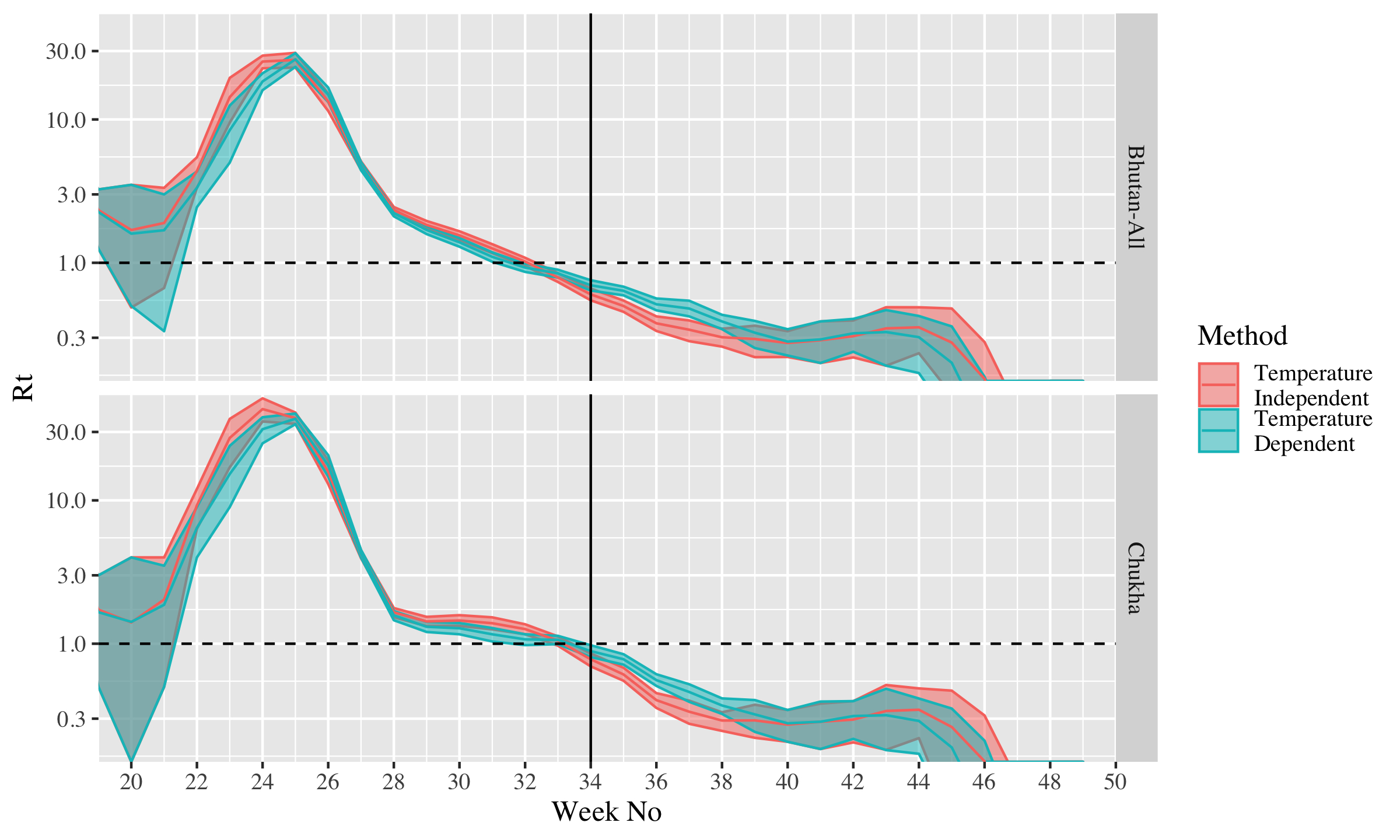

Supplement: Supplementary file 1 [file ijerph-18-00354-s001.zip › Supplementary Figure S7.tiff]

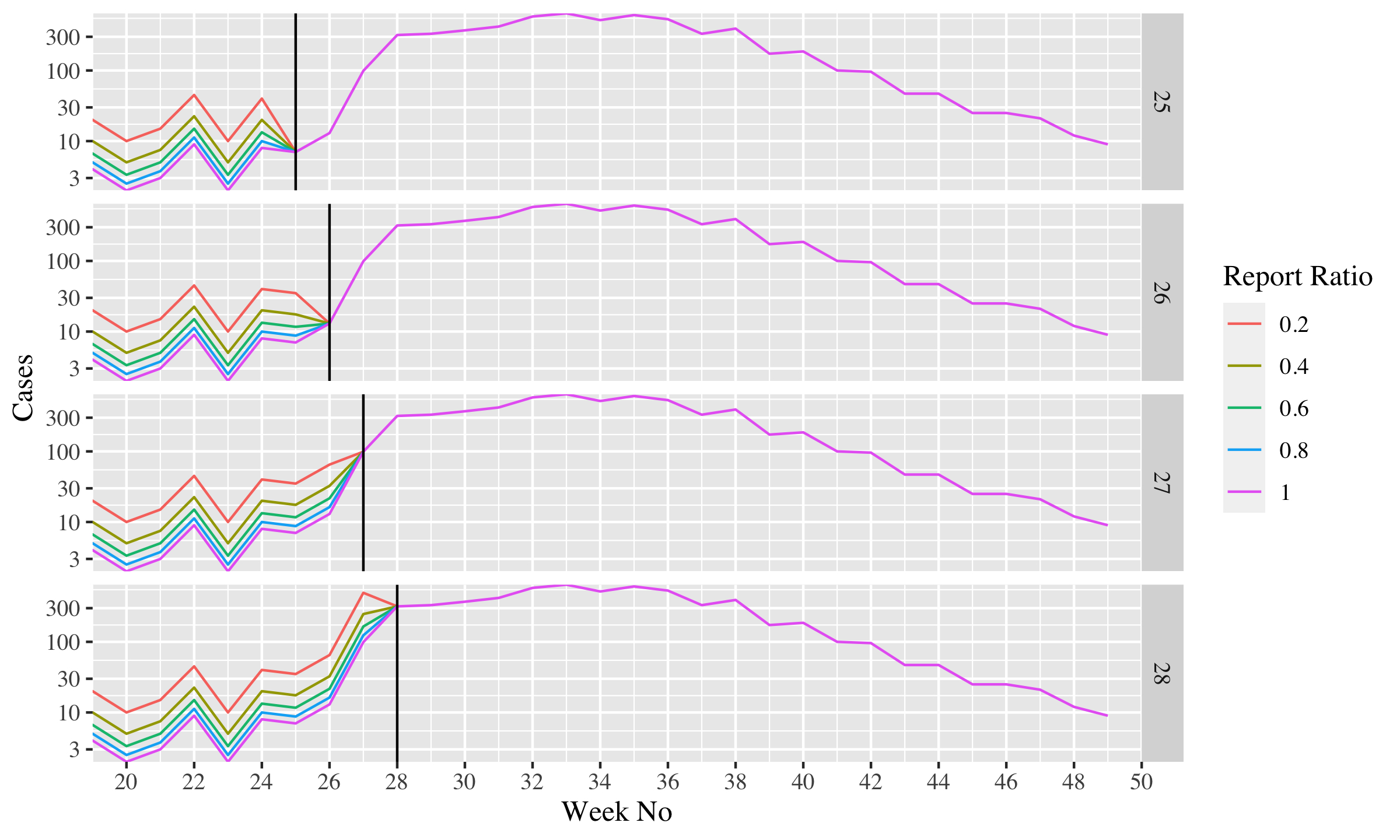

Supplement: Supplementary file 1 [file ijerph-18-00354-s001.zip › Supplementary Figure S8.tiff]

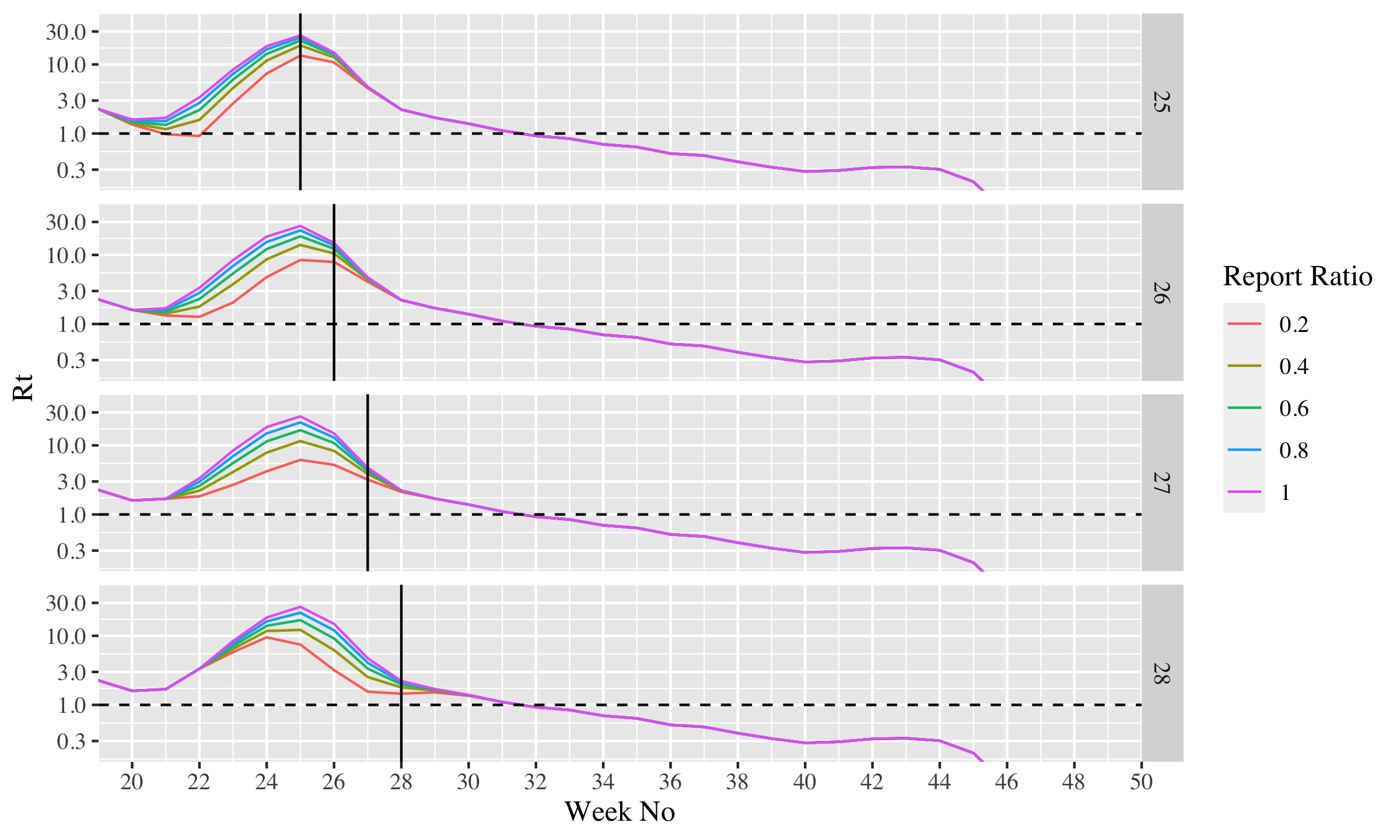

Supplement: Supplementary file 1 [file ijerph-18-00354-s001.zip › Supplementary Figure S9.tiff]
